# *In Vivo* and *In Silico* Evaluation of a Polyphenol Fraction Extracted From *Argania spinosa* L. Press‐Cake Against Ethylene Glycol‐Induced Calcium Oxalate Urolithiasis in Rats: Biochemical, Histopathological, and Molecular Modeling Analyses

**DOI:** 10.1002/fsn3.71238

**Published:** 2025-11-20

**Authors:** Fatima Ezzahra El Oumari, Anouar Hmamou, Amal Elrherabi, Naima Mammate, Mohamed El Fadili, Hinde El Fatmi, Salim Belchkar, Fahd A. Nasr, Mohammed Al‐zharani, Ashraf Ahmed Qurtam, Dalila Bousta, Tarik Sqalli Houssaini

**Affiliations:** ^1^ Laboratory of Epidemiology and Research in Health Sciences, Faculty of Medicine, Pharmacy and Dental Medicine University of Sidi Mohammed Ben Abdellah Fez Morocco; ^2^ Laboratory of Engineering, Electrochemistry, Modeling and Environment, Faculty of Sciences Dhar El Mahraz Sidi Mohamed Ben Abdellah University Fez Morocco; ^3^ Laboratory of Bioresources, Biotechnology, Ethnopharmacology and Health, Faculty of Sciences University Mohammed 1st Oujda Morocco; ^4^ Engineering Materials, Modeling and Environmental Laboratory, Faculty of Sciences Dhar El Mehraz Sidi Mohammed Ben Abdellah University Fez Morocco; ^5^ Laboratory of Anatomical Pathology Analysis University of Hospital Hassan II Fez Morocco; ^6^ Biology Department, College of Science Imam Mohammad Ibn Saud Islamic University (IMSIU) Riyadh Saudi Arabia; ^7^ Morocco Laboratory of Biotechnology, Environment, Agri‐Food, and Health (LBEAS), Faculty of Sciences Dhar El Mahraz University of Sidi Mohammed Ben Abdellah Fez Morocco

**Keywords:** *Argania spinosa* L, crystalluria, hepatotoxicity, molecular docking, nephrotoxicity, polyphenols, urolithiasis

## Abstract

In Morocco, *Argania spinosa* L. is traditionally used to treat kidney‐related disorders such as urolithiasis. However, its anti‐urolithiatic potential remains poorly documented from a scientific standpoint. This study aimed to evaluate the anti‐crystallization, nephroprotective, and hepatoprotective effects of a polyphenol‐rich fraction from *A. spinosa* press cake in an ethylene glycol‐induced urolithiasis rat model, and to explore its pharmacokinetic profile and molecular interactions through *in silico* analyses. Urolithiasis was induced in male Wistar rats by administering 0.75% ethylene glycol in drinking water for 15 days. Treatment groups received either the polyphenol‐rich fraction or cystone. Biochemical analyses of urine and blood were performed, and renal and hepatic tissues were subjected to histopathological examination. *In silico* studies included molecular docking and ADMET (absorption, distribution, metabolism, excretion, and toxicity) predictions to evaluate pharmacokinetic properties and target interactions. Ethylene glycol administration led to a significant increase in urinary pH and protein levels, reduced urinary magnesium, and caused renal and hepatic tissue damage. Treatment with the polyphenol‐rich fraction significantly corrected these alterations (*p* < 0.05), reduced histological lesions, and restored biochemical parameters. Molecular docking revealed high binding affinities between the major polyphenols and targets involved in urolithiasis pathogenesis. ADMET analyses suggested favorable pharmacokinetic profiles and low toxicity. The polyphenol‐rich fraction of *A. spinosa* press cake shows promising protective effects against ethylene glycol‐induced kidney stone formation and associated organ toxicity. These findings highlight its potential as a natural therapeutic agent for the prevention and management of urolithiasis and renal dysfunction.

## Graphical abstract

Molecular docking of identified polyphenols from argan press‐cake on 3RQ4 protein, and in vivo investigation of anti‐urolithiatic activity in rats including biochemical and histological analyses.

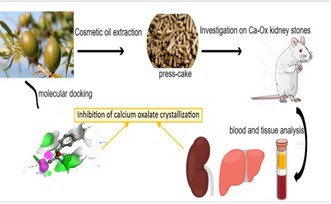

**Abbreviations**
ADMET: absorption, distribution, metabolism, excretion, toxicity
ALT: alanine transaminase
AST: aspartate transaminase
BBB: blood brain barrier
Ca OX: calcium oxalate
Ca OX: calcium oxalate
CNS: central nervous system
EG: ethylene glycol

## 1 Introduction

About 12% of the population develops urinary stones, with recurrence rates of 70%–80% in men and 47%–60% in women. Urinary stones typically form as blockages and can cause serious complications, including bleeding and infections that require intensive medical attention. The medical community is increasingly concerned about their widespread occurrence and rising global prevalence (Baheti and Kadam 2013; Bano et al. 2018; Delbarre et al. 2023; Filho et al. 2025; Meria et al. 2023). In addition to microscopic or gross hematuria, ureteric obstruction, and urinary tract infection, kidney stones may cause severe pain or, in some cases, no symptoms at all. They are also associated with other manifestations such as malaise, cloudy or foul‐smelling urine, burning during urination, nausea, vomiting, fever, and chills (Akram et al. 2024; Siddiqui et al. 2018). Hyperoxaluria is one of the key factors contributing to calcium oxalate stone formation in humans and plays a central role in lithogenesis. It is defined as a condition characterized by an excessive concentration of oxalate in the urine, resulting in a solution that contains more solute than can normally be dissolved (Demoulin et al. 2022; Groothoff et al. 2023; Mandrile et al. 2023). The cost of current treatments is often beyond the reach of the average person, and recurrence is common, requiring years of careful patient follow‐up. Therefore, there is great potential in the search for antilithiatic agents derived from natural sources that are both effective and free from adverse effects. The chronic use of synthetic drugs and their associated side effects have driven growing interest in alternative therapies that are potent, safe, and natural (Bano et al. 2018). The effectiveness of phytotherapeutic agents as adjunct or alternative therapies for the treatment of urolithiasis has been demonstrated by evidence from numerous previous studies (Dhar and Shoskes 2007; Nirumand et al. 2018; Rocha and Granato 2021). The press cake derived from the indigenous tree *Argania spinosa* L. was evaluated in vitro at concentrations of 0.25 and 0.5 mg/mL for its ability to inhibit calcium oxalate crystallization. This species is well known for its numerous biological benefits (El Oumari, Mammate, et al. 2022). Regarding urolithiasis brought on by calcium oxalate crystals, the total extract and its components show significant promise (El Oumari, Bousta, et al. 2022; El Oumari et al. 2021). Numerous studies have shown the pharmacological effects of *A. spinosa* L., including its potential to prevent and cure chronic diseases as well as its many biological features, including antibacterial, antioxidant, antihyperglycemic, anti‐tumor, anti‐proliferative, and insecticidal (Benabdesslem et al. 2022; Idrissi et al. 2023; Lfitat et al. 2023; Mechqoq et al. 2021; Mourjane et al. 2023, 2022; Naama et al. 2023). Other previous studies have also noted the presence of various phytochemicals in press cake, including catechol, tyrosol, catechin, and epicatechin, with numerous pharmacological activities (Kamal et al. 2021). Regarding the identified compounds in the polyphenolic fraction, in our previous study, we reported that this fraction contains isocyanic acid and p‐hydroxybenzoic acid as major compounds (El Oumari, Mammate, et al. 2022).

In Morocco, the *A. spinosa* L. has long been utilized by the local population to address various health issues, ranging from digestive problems to skin ailments, skeletal disorders, circulatory issues, and urinary stones (El‐Hilaly et al. 2003; Ouhaddou et al. 2014). In this context, our study presents novel findings on the anti‐urolithiasis properties of the polyphenolic fraction extracted from the press cake of *A. spinosa* L. against calcium oxalate stones, utilizing the male Wistar rat model. Additionally, our current investigation aims to evaluate the toxicity profiles of the molecules identified within this polyphenolic fraction extracted from *A. spinosa* L. oil cake (El Oumari, Mammate, et al. 2022) employing *in silico* ADMET toxicity prediction methods. Furthermore, we seek to establish associations between these compounds and a specific targeted protein, 3RQ4A, known to interact with calcium oxalate crystals (Aggarwal et al. 2013).

## 2 Material and Methods

### 2.1 Plant Material

The fruits of *A. spinosa* L., collected in the Agadir region (30°25′ N, 9°36′ W, Morocco), were pulped and peeled prior to use. The nuts were cracked to obtain kernels, which were subsequently broken to extract cosmetic oil. The resulting *A. spinosa* L. press cake was dried at 25°C and ground into a fine powder. The specimens have been deposited in the herbarium under the reference numbers “BPRN67” for *A. spinosa* (L.) Skeels.

### 2.2 Polyphenols Extraction

With minor modifications, 1–2 g of the plant powder were added with 40 mL of each solvent extraction (ethanol, acetone, and methanol 70%), and was left to infuse for 30 min. Filtered marcs were used after a 20‐min centrifugation at 4000 rpm the mixed filtrates were evaporated and kept at 4°C until any further evaluation (Souhila et al. 2013).

### 2.3 Experimental Animals

Healthy male Wistar rats (150–200 g) were used in this study, following the procedure of (Saleem et al. 2020). The animals were obtained from the animal facility of the Faculty of Medicine and Pharmacy of Fez, housed in metabolic cages, and acclimatized for 3 days prior to experimentation. All experimental procedures complied with internationally recognized ethical standards, including the European Directive 86/609/EEC and the NIH publication No. 85–23 (revised 1985) (Hmamou et al. 2024). The study protocol was approved by the Institutional Ethics Committee for the Care and Use of Laboratory Animals at the Faculty of Medicine and Pharmacy of Fez, Sidi Mohamed Ben Abdellah University (approval no. 05/2023/LERHS).

### 2.4 In Vivo Evaluation of Anti‐Urolithiatic Activity

**FIGURE 1:**
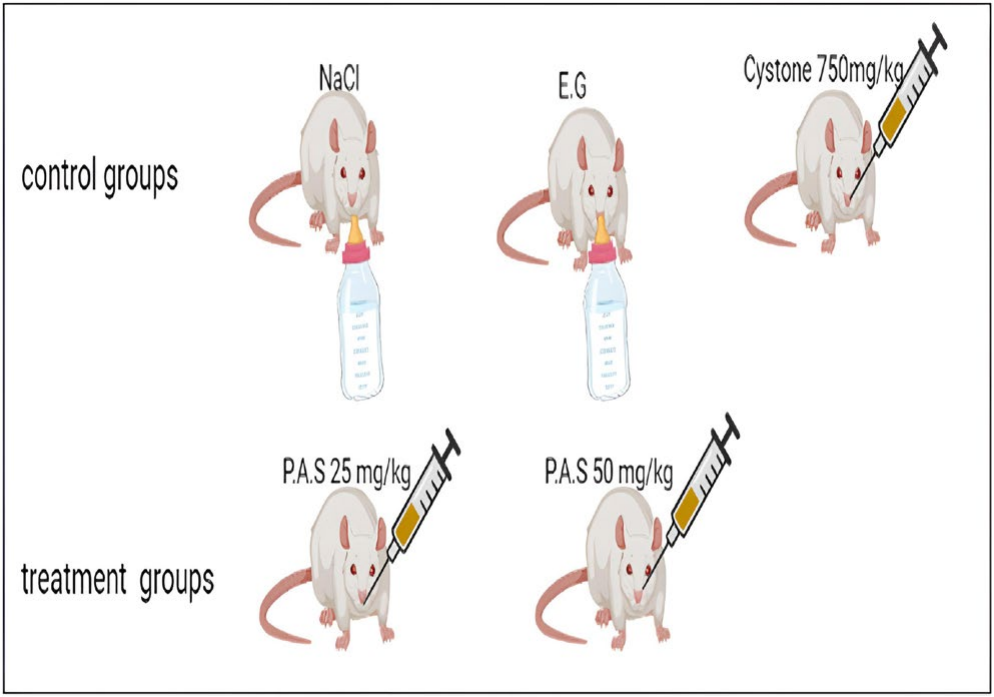
Experimental protocol for in vivo anti‐urolithiatic activity.

Before any induction, the rats were placed in metabolic cages to collect urine samples for microscopic analysis. Only animals with no crystals observed in their urine were selected for the study. The number of animals used was *n* = 6 per cage. Urolithiasis was induced by administering ethylene glycol (0.75% v/v) in drinking water for 15 days. Group I, the healthy control, received only the vehicle. Group II served as the disease control and received ethylene glycol alone for 15 days. Group III, the positive control, received ethylene glycol for 15 days followed by Cystone (750 mg/kg orally) for 2 weeks. Groups IV to V received ethylene glycol for 15 days, then were treated with polyphenol fractions from Argan press cake at doses of 25 and 50 mg/kg for an additional 15 days (Figure 1).

**FIGURE 2:**
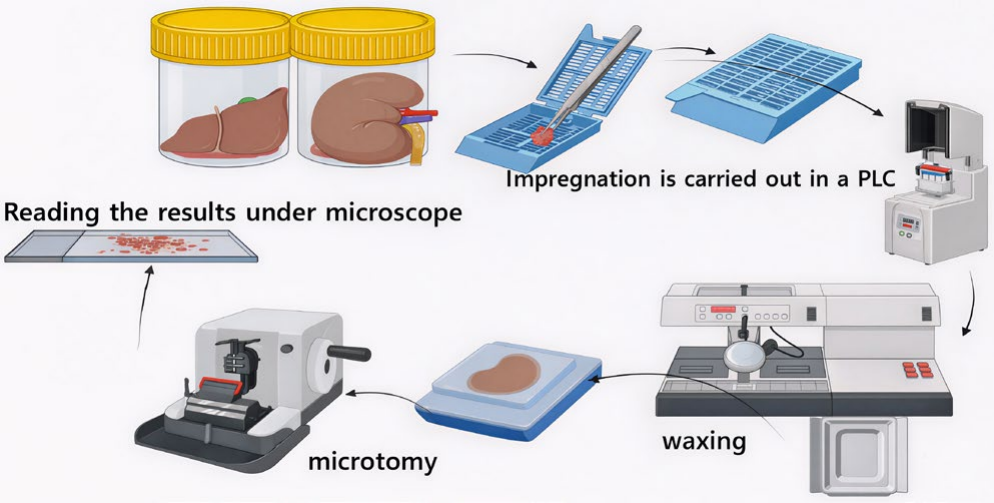
Anatomical pathology methodology used for tissue analysis.

At the end of the experiment, rats were kept in metabolic cages, and urine samples were taken after 24 h in an ice bath. 1 mL of the collected urine samples was used for a crystalluria test to observe crystals, and the animals were sacrificed. Blood was drawn from each rat to measure biochemical factors such as creatinine, urea, hepatic enzymes ASAT, and ALAT. For the anatomical pathology method, the animals' kidneys and liver were removed, and rinsed out in PBS (pH 7.4). The tissues were placed in paraffin after being fixed in 10% neutral buffered formalin. Using a microtome, sections with an average thickness of 4 mm were cut and placed on slides. Hematoxylin and Eosin stained the slides for histopathological analysis (Figure 2). Then, the slides were photographed on camera using a microscope.

### 2.5 ADMET *In Silico* Prediction and Molecular Docking Simulation

#### 2.5.1 ADMET *In Silico* Prediction

The prediction of pharmacokinetic properties of absorption, distribution, metabolism, excretion, and toxicity (ADME‐Tox) was done with the help of Swiss ADMET and pk CSM servers (Daina et al. 2017). The BOILED‐Egg model based on the calculation of lipophilicity given by the logarithm of the partition coefficient between n‐octanol and water (Log PO/W) and polarity marked by the topological polar surface area (TPSA) of small molecules was applied to identify the potent central nervous system (CNS) agents, with the highest probability to cross the blood–brain barrier (BBB) (El Fadili, Er‐Rajy, Imtara, et al. 2022; El Fadili, Er‐Rajy, Kara, et al. 2022). C1, C2, C3, C4, and C5 are the following compounds respectively: isocyanic acid, isobutyric acid, hydroxybenzoic acid, catechol, and ephedrine.

#### 2.5.2 Molecular Docking Simulation

With the help of the Protein Data Bank (PDB), we have extracted 3RQ4 encoded protein applying the method of X‐RAY diffraction, for a good resolution of 1.80 Å. Thereafter, we started the AutoDock 4.2 software's docking computations (Norgan et al. 2011). By centralizing the grid box and placing the sizes 100, 100, and 100 in its three‐dimensional structure with a spacing of 0.375 Å, we ran 10 genetic algorithms totaling 25 million evals with the help of the AUTOGRID algorithm. Lastly, using Discovery Studio 2021, we were able to visualize the protein‐ligand complex's 2D (left) and 3D (right) interactions after obtaining the strongest complex out of the generated conformations.

## 3 Results

### 3.1 Chemical Profile of Polyphenol Fractions From *A. spinosa* L.

In the present study, we used the same polyphenol fraction sample extracted from *A. spinosa* L. press cake as in our recent study (El Oumari, Mammate, et al. 2022). In that previous investigation, the evaluation was limited to an in vitro calcium oxalate crystallization assay. In contrast, the current research expands the approach by assessing the in vivo anti‐urolithiatic potential of this fraction. GC–MS analysis confirmed the presence of five major compounds: isocyanic acid, isobutyric acid, p‐hydroxybenzoic acid, catechol, and ephedrine. These findings highlight the chemical richness of the extract and support its biological potential in the prevention and treatment of urolithiasis.

### 3.2 Anti‐Urolithiatic Activity Evaluation

#### 3.2.1 Effect on Urine Volume and pH

Rats' urine volume significantly increased (*p <* 0.001) after receiving 0.75% (v/v) EG. At the end of the experiment, urinary output was 6.2 ± 1.15 mL/d in the control group and 9 ± 0.45 mL/d in the EG group. Administration of fractions during the experiment further caused a higher production of urine compared with cystone and EG groups. Compared to the control group, the EG group's urine showed a higher pH level. However, cystone and fractions did not significantly alter the pH of the urine (Table 1).

**TABLE 1:** Urinary pH values and urinary volumes measured after urine collection from metabolic cages.

Tested groups	pH value	Urinary volume (mL/24 h)
NaCl	6.54 ± 0.27	6.2 ± 1.15
Ethylene glycol	8.21 ± 1.13	9 ± 0.45
Cystone	7.06 ± 0.82	6.8 ± 0.69
A.S 50 mg/mL	6.72 ± 0.44	9 ± 2.2
A.S 25 mg/mL	6.87 ± 0.52	10.5 ± 1.75

#### 3.2.2 Crystalluria Analysis

**FIGURE 3:**
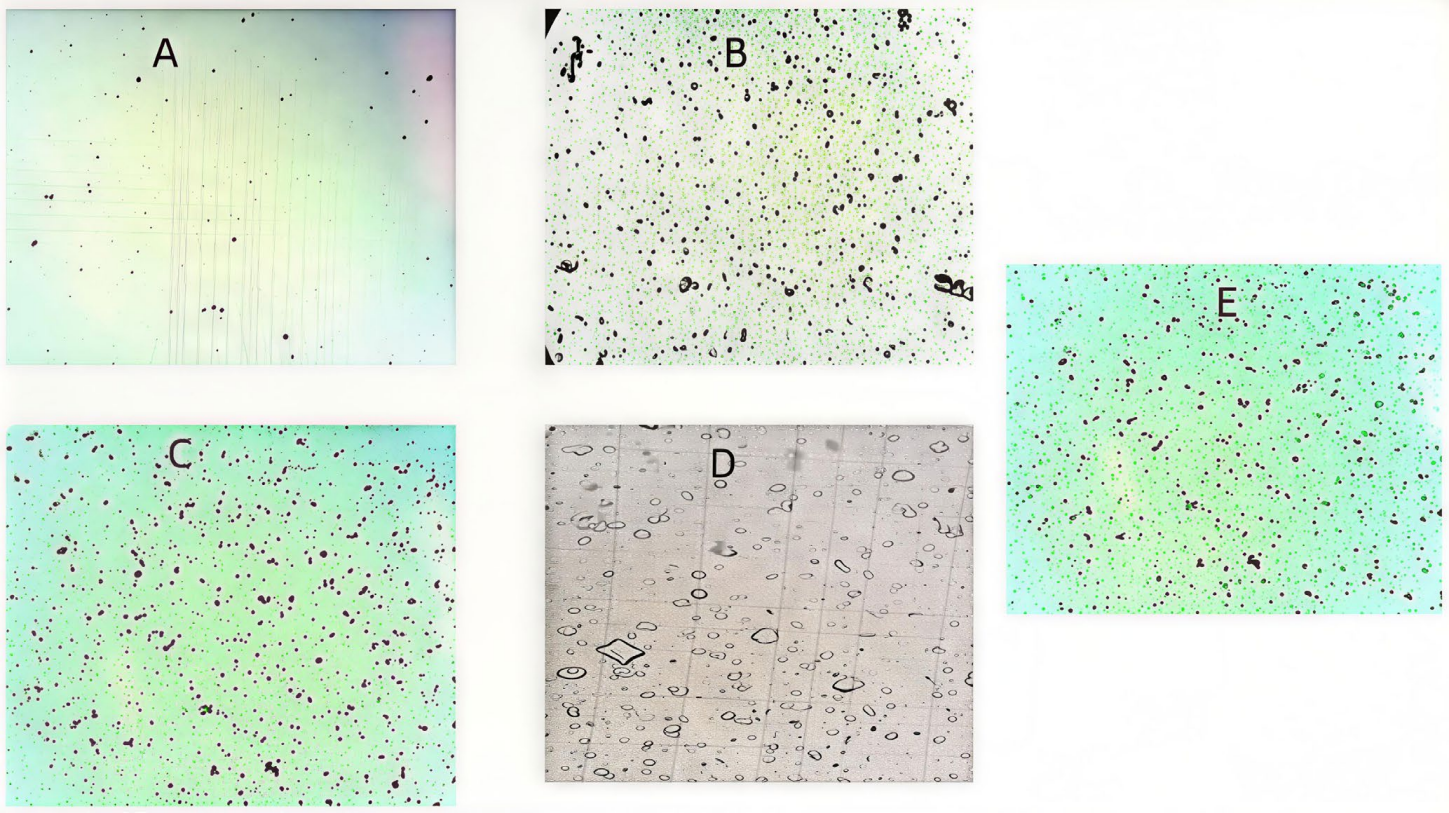
Result of crystalluria after treatment with polyphenols from *A.S* press‐cake. (A) NaCl; (B) ethylene glycol; (C) cystone; (D) polyphenol at a dose of 50 mg/kg; (E) polyphenol at a dose of 25 mg/kg.

Microscopic observation of urine samples revealed a decrease in the number and size of calcium oxalate crystals in the groups treated with polyphenolic fraction. The observation of the urine from the treated group with polyphenolic fraction at a dose of 50 mg/kg showed that it was the most effective treatment with the transformation of whewellite to weddellite crystals (Figure 3).

#### 3.2.3 Serum Biochemical Parameters (Renal Profile)

Table 2 displays the impact of tested fractions on serum parameters. In the EG group, an increase in creatinine, urea, and calcium was observed. Simultaneously significant reductions in serum creatinine, urea, and calcium were observed in the treated groups with fractions and cystone, except for the treated group with A.S P‐C at a dose of 50 mg/kg. Whereas, the magnesium level increased in all treated groups to normal value.

**TABLE 2:** The effect of tested fractions on serum parameters for renal profile after 15 days of treatment.

Renal profile	Normal control	Ethylene glycol	Cystone	A.S 25 mg/kg	A.S 50 mg/kg
Creatinine (mg/dL)	0.858 ± 0.32	1.55 ± 0.54	0.98 ± 0.95	0.82 ± 0.22	1.32 ± 0.25
Urea (mg/dL)	36.32 ± 2.12	49.32 ± 1.5	37.5 ± 0.35	44.5 ± 0.55	49.4 ± 0.64
Magnesium (mg/dL)	2.65 ± 0.32	1.54 ± 0.85	2.32 ± 0.15	1.96 ± 0.25	1.45 ± 0.03
Calcium (mg/dL)	9.35 ± 0.02	14.2 ± 0.01	10.5 ± 0.05	11.8 ± 0.03	10.02 ± 0.02

#### 3.2.4 Serum Biochemical Parameters (Hepatic Profile)

For the hepatic profile (Table 3), ALT and AST levels increased significantly (*p <* 0.05) in the EG group compared to the control. Total protein showed no statistically significant change in treated groups.

**TABLE 3:** The effect of tested fractions on serum parameters for hepatic profile after 15 days of treatment.

Hepatic profile	Control	Cystone	Ethylene glycol	A.S 25 mg/kg	A.S 50 mg/kg
AST (UI/L)	95.65 ± 1.35	94.5 ± 1.5	120.5 ± 3.41	97.5 ± 2.8	101.2 ± 2.1
ALT (UI/L)	65.2 ± 0.85	65.8 ± 0.7	85.2 ± 0.25	64.9 ± 1.76	67.8 ± 3.41
Total protein (g/L)	46.2 ± 2.12	45.5 ± 1.9	47.9 ± 1.7	44.8 ± 0.95	45.58 ± 1.54

#### 3.2.5 Histopathological Study of Kidneys

**FIGURE 4:**
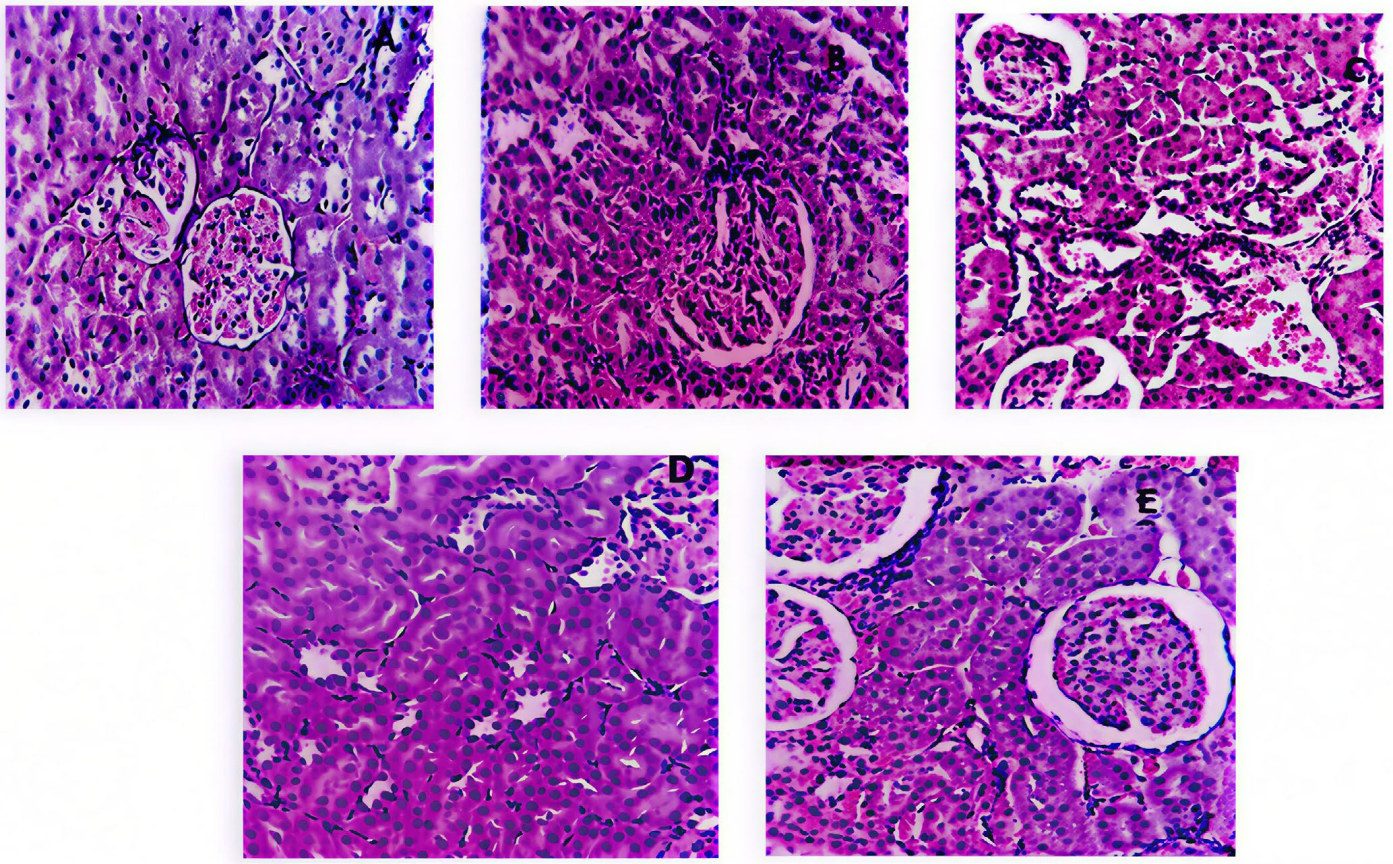
Effect of polyphenol fractions on the anatomical histology of the kidney. (A) Photomicrographs of the renal sections of NaCl group. (B) Photomicrographs of the renal sections of ethylene glycol group. (C) Photomicrographs of the renal sections of Cystone group. (D) Photomicrographs of the renal sections of A.S at 25 mg/kg. (E) Photomicrographs of the renal sections of A.S at 50 mg/kg (magnification ×10).

The nephron segment of the treated groups did not exhibit any abnormalities or calcium oxalate deposits, as determined by histopathological investigation of the kidneys. In other ways, calcium oxalate deposits inside the tubules, dilatation of the proximal tubules, interstitial inflammations, and acute microglomerular focus were identified in the renal tissue of urolithiatic rats (Ethylene glycol group) (Figure 4).

#### 3.2.6 Histopathological Study of Liver

**FIGURE 5:**
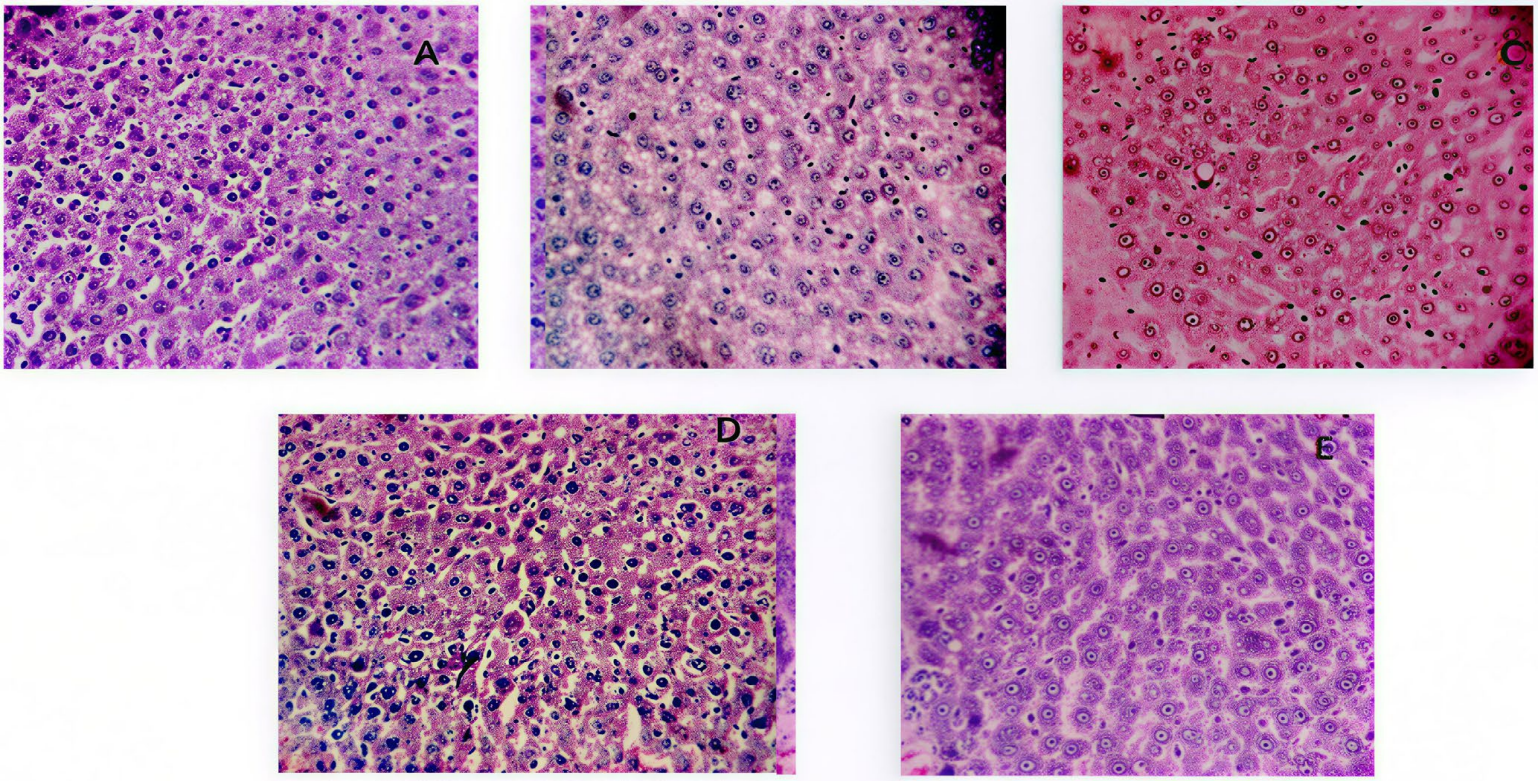
Effect of polyphenol fractions on the anatomical histology of the liver. (A) Photomicrographs of the liver section of the NaCl group. (B) Photomicrographs of the liver sections of the ethylene glycol group. (C) Photomicrographs of the liver section of the Cystone group. (D) Photomicrographs of the liver section of *A.S* at 25 mg/kg. (E) Photomicrographs of the liver section of *A.S* at 50 mg/kg (magnification ×10).

The administered amounts were nontoxic for liver anatomical pathology, and microscopic examination of liver sections in all treated groups showed no abnormalities, with the exception of the ethylene glycol group, which displayed a slight acute injury in liver tissue (Figure 5).

### 3.3 ADMET *In Silico* Prediction and Molecular Docking Simulation

The ADMET prediction reported (Table 4) that all five molecules have good human intestinal absorption (HIA superior to 74%), good distribution defined by permeability to the blood–brain barrier (BBB) superior to −1 Log BB, and permeability to the central nervous system (CNS) included between −2 and −3 Log PS. They present no inhibitory effect on the cytochromes. According to the AMES test, all five molecules are predicted as non‐toxic inhibitors, but the compound C3 presents a positive Hepatotoxicity effect and C1, C2, and C3 present skin Sensitization. Thus, C4 and C5 were predicted as non‐toxic inhibitors penetrating the central nervous system (CNS) without any toxicity.

**TABLE 4:** ADMET *in silico* pharmacokinetic properties of *Argania spinosa* press‐cake polyphenol fraction.

Compounds number	Absorption	Distribution		Metabolism							Excretion	Toxicity		
	Intestinal absorption (human)	BBB permeability	CNS permeability	Substrate		Inhibitor					Total clearance	AMES toxicity	Hepatotoxicity	Skin sensitization
				Cytochromes										
				2D6	3A4	1A2	2C19	2C9	2D6	3A4				
	Numeric (% absorbed)	Numeric (log BB)	Numeric (log PS)	Categorical (yes/no)							Numeric (log ml/min/kg)	Categorical (yes/no)		
C1	80.118	−0.278	−2.12	No	No	No	No	No	No	No	0.151	No	No	Yes
C2	91.072	−0.276	−2.341	No	No	No	No	No	No	No	0.318	No	No	Yes
C3	92.004	0.146	−1.995	No	No	No	No	No	No	No	0.948	No	Yes	Yes
C4	74.377	−0.331	−2.91	No	No	No	No	No	No	No	0.666	No	No	No
C5	94.469	−0.378	−2.638	No	No	No	No	No	No	No	0.766	No	No	No

**FIGURE 6:**
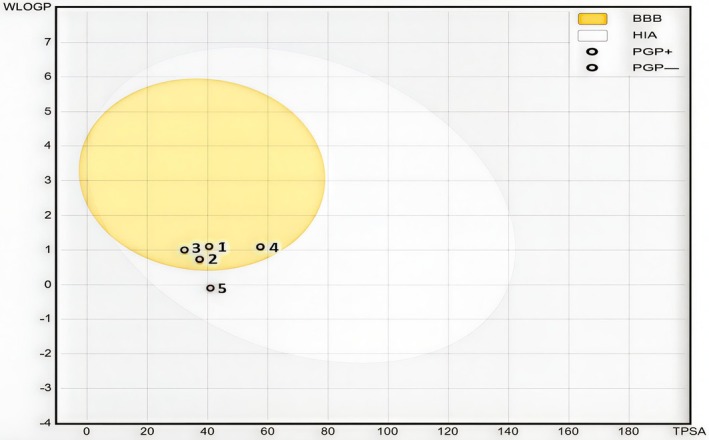
Boiled‐eggs model of *Argania spinosa* press‐cake (polyphenol fraction).

The BOILED‐Egg (Figure 6) as an accurate predictive model shows that all molecules are part of the yellow Egan egg, except the compound C5 which is part of the white Egan egg. So, C1, C2, C3, and C4 were predicted to passively permeate through the blood–brain barrier (BBB). Inversely, C5 was predicted to be passively absorbed by the gastrointestinal tract. Therefore, the most active molecule marked as “C4” was successfully predicted as a central nervous system agent with a good ADME‐Tox profile.

**FIGURE 7:**
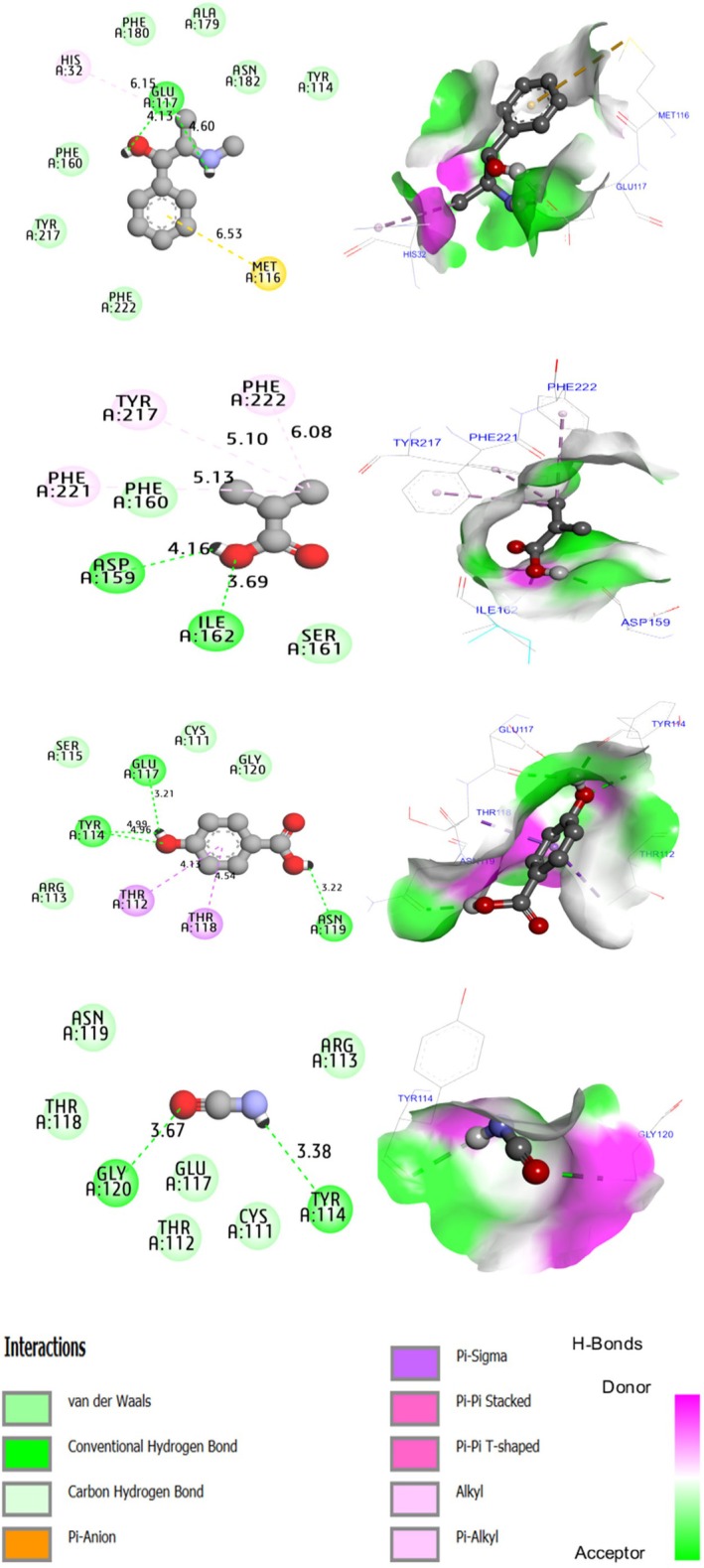
2D and 3D views of intermolecular interactions produced between C1, C2, C3, C4 ligands toward 3RQ4.pdb encoded protein, with binding energies of −4.600, −4.21, −4.96 and 4.21 kcal/mol, respectively.

The results of molecular docking (Figure 7) performed between the targeted protein encoded as 3RQ4.pdb and five active molecules (C1, C2, C3, C4, and C5) demonstrate that: nC1 generated a hydrogen bond toward Glu 117 amino acid and created a chemical binding with His 32. C2 produced a chemical bound of Pi‐Alkyl type with PHE 222 amino acid. Two H‐bonds were produced with Asp 159 and Il162 amino acids. C3 formed a hydrogen bond toward His 183 amino acid and created a chemical bound of Pi‐Pi stacked type with Tyr 114 and PHE 222 amino acid. C4 produced three hydrogen bonds toward Glu 117, Tyr 114, and Asn 119 amino acids. C5 formed two hydrogen bonds toward Tyr114 and Glu120 amino acids. Considering that chemical bonds of H‐bonds make the ligand (active molecule) so stable toward the amino acid's residues of the responsible protein.

### 3.4 Docking Validation Protocol

**FIGURE 8:**
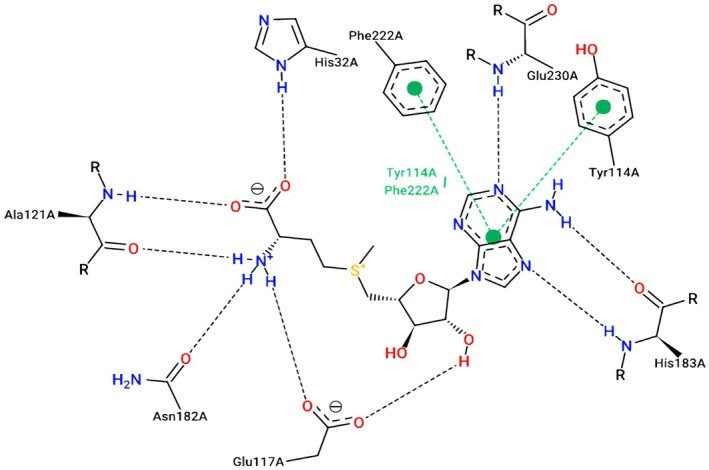
The active sites of co‐crystallized ligand complexed with 3RQ4 protein.

All active molecules were docked in the targeted protein's active sites, indicating that the molecular docking process was successful for the 3RQ4.pdb code, which was obtained experimentally in Figure 8. Where Phe222, His32, Tyr 114, His183, Glu117 all resulted in the (C1, C2, C3, C4, and C5—3RQ4 protein) complexes.

## 4 Discussion

The anti‐urolithiatic activity in vivo was studied to evaluate the efficacy of polyphenolic fraction from *A. spinosa* press cake. Ethylene glycol (EG) intake in rats has been frequently utilized as an experimental model for studying the anti‐urolithiatic agents. However, when EG is administered alone, kidney crystal deposition can be very variable. To produce a uniformly high rate of kidney crystal deposition, male rats were given with 0.75% ethylene glycol, which demonstrated deposition of calcium oxalate (CaOx) crystals in the kidney after 7 days (Fan et al. 1999). According to reports, non‐specific dehydrogenase oxidizes EG to oxalic acid, which causes hyperoxaluria, a significant contributor in urolithiasis. EG transformed to CaOx monohydrate, which induces renal mitochondrial toxicity, identical to clinical CaOx renal calculi (McMartin and Wallace 2005).

Large and numerous crystals were found in the urine of untreated mice 15 days after. A similar conclusion was obtained in numerous research (Fan et al. 1999; McMartin and Wallace 2005). However, after 15 days of therapy with polyphenolic fraction, urinary crystals were dramatically decreased (*p <* 0.001) at both low and high doses. This effect may help prevent stone formation by excretion of small particles and reducing their urinary tract retention chances.

Urinary magnesium (Mg) inhibits stone formation after binding with oxalate, it decreases oxalate availability to bind with calcium. The reason why, in both stone formers and stone‐forming rats, magnesium levels are low (Soundararajan et al. 2006). After treatment Mg levels returned to normal. Animals treated with large doses of tested fractions showed an important increase (*p* < 0.01) in Mg levels in the urine. The data demonstrate the efficacy of the tested fraction in lowering the magnesium level and preventing stone formation. The markers of renal and tubular injury are creatinine and urea (Patel et al. 2012). Our result is similar to this study of Patel et al. (2012) and showed an increase in both serum parameters for the EG group in comparison with the other groups.

The serum creatinine level of ethylene glycol group, was increased with 1.55 ± 0.54 after the ethylene glycol administration (orally) till day 14. In the negative control group the serum creatinine level is 0.858 ± 0.32. The animals treated with standard drug (Cystone 750 mg/kg), and fraction (25 mg/kg), only were observed that significant decreased in serum creatinine level with 0.98 ± 0.95 and 0.82 ± 0.22 mg/dL respectively compared to ethylene glycol group.

The serum urea level of the negatif control is 36.32 ± 2.12. In the ethylene glycol group, and the group treated with A.S press‐cake (50 mg/kg), the serum urea level increased to the maximum measurable value of 49.32 ± 1.5 and 49.4 ± 0.64 mg/dL respectively. Whereas the urea level of cystone and A.S press‐cake (25 mg/kg) was 37.5 ± 0.35 and 44.5 ± 0.55 mg/dL respectively.

The serum calcium level of all experimental groups, except normal control group, was decreased significantly after the ethylene glycol administration (orally). In the negative control group, the serum calcium level decreased to the maximum measurable value of 9.35 ± 0.02 mg/dL and found to be significant decreased (*p <* 0.001). In ethylene glycol animals, serum calcium level is 14.2 ± 0.01 mg/dL. Polyphenols at a dose of 25 mg/kg normalized the calcium level.

The aforementioned conclusions were corroborated by the histopathological characteristics. No revealed calcium oxalate deposits or other abnormalities were in the nephron segment of the vehicle treatment groups. Conversely, the renal tissue of urolithiatic rats (ethylene glycol group) showed multiple calcium oxalate deposits inside the tubules, dilatation of the proximal tubules, interstitial inflammations, and acute microglomerular focus. For the anatomical analysis of livers, the given doses were non‐toxic, and microscopic analysis revealed no abnormalities in liver sections in all treated groups. Expect the analysis of the ethylene glycol group, which showed a mild acute injury in liver tissues. According to studies, high oxalate exposure damages cells by causing membrane lipid peroxidation and the production of intracellular reactive oxygen species. Therefore, decrease in renal oxidative stress could be a beneficial method in the treatment of urolithiasis (Bano et al. 2018).

Recent studies suggest that histone methyltransferase was overexpressed in renal tubules of patients with diabetic nephropathy, indicating that the expression correlates with the development of chronic kidney disease (Yu and Zhuang 2019). However, hyperglycemia decreased expression in type 1 diabetes‐associated renal fibrosis (Miao et al. 2014).

Polyphenols from *A.S* Press‐cake constituents were subjected to docking studies with 3RQ4 encoded protein. The molecular docking simulations clearly show from both 2D and 3D visualizations that the major compounds of polyphenolic fraction of *A. spinosa* press‐cake (El Oumari, Mammate, et al. 2022) named isocyanic acid, isobutyric acid, hydroxybenzoic acid, and catechol labeled C1, C2, C3, C4 respectively were complexed to 3RQ4 encoded protein producing various intermolecular interactions with lowest binding energies of −4.600, −4.21, −4.96 and −4.21 kcal/mol, respectively. Therefore, the anti‐urolithiatic activity can be explained by detecting chemical bonds amino acids of targeted protein. However, the interaction between targeted protein and major compounds should be proven by experimental assays and experimental validation.

The polyphenol fraction from *A. spinosa* L. press cake demonstrated significant protective effects against ethylene glycol‐induced urolithiasis in rats, improving biochemical parameters, reducing histopathological damage, and exhibiting promising interactions with relevant molecular targets *in silico*. These results support its potential as a natural therapeutic agent for preventing and managing kidney stone formation and associated organ dysfunction. However, this study has some limitations. The study focused on a single urolithiasis induction model and limited dosing regimens, which may not capture the full spectrum of the fraction's efficacy or safety. Finally, while *in silico* analyses provided insights into pharmacokinetics and molecular interactions, in vivo pharmacokinetic and long‐term toxicity studies are still needed to confirm its therapeutic potential. Future research should address these limitations by including diverse animal models, dose optimization, and clinical validation.

## 5 Conclusion

In conclusion, EG demonstrated increased excretion of minerals and crystals implicated in the formation of urinary calculi in the urine, as well as hepato‐renal toxicity. The application of polyphenols from *A. spinosa* L. press‐cake, at a dose of 25 mg/kg, significantly reduced these toxicities. Therefore, the results might pave the way to use those polyphenols in the prevention and/or management of urinary calculus, renal damage, and chronic toxicity. Because of their excellent pharmacokinetic features, polyphenol compounds are attractive candidates for the creation of pharmacological medicines, according to the ADMET study. The docking simulation showed that the major compounds were complexed to targeted protein producing various intermolecular interactions. However, assays that are more experimental are still necessary for the validation of polyphenols' safety and efficacy such as sub‐acute toxicity and in vitro assays as well as clinical trials.

## Author Contributions

**Fatima Ezzahra El Oumari:** conceptualization, resources, data curation, formal analysis, investigation, methodology, software, supervision, visualization, writing original draft, and writing review and editing. **Anouar Hmamou:** methodology and visualization. **Amal Elrherabi:** visualization, writing original draft, and writing review and editing. **Naima Mammate:** methodology, data curation, and formal analysis. **Mohamed El Fadili:** formal analysis and software. **Hinde El Fatmi:** data curation. **Salim Belchkar:** data curation. **Fahd A. Nasr:** funding acquisition, visualization, and writing review and editing. **Mohammed Al‐Zharani and Ashraf Ahmed Qurtam:** writing – review and editing and visualization. **Dalila Bousta and Tarik Sqalli Houssaini:** resources and writing review and editing. All authors have read and agreed to the published version of the manuscript.

## Ethics Statement

All animal experiments were conducted in accordance with internationally recognized ethical standards, including the European Directive 86/609/EEC and the National Institutes of Health (NIH) Guide for the Care and Use of Laboratory Animals (NIH Publication No. 85–23, revised 1985). The study protocol was reviewed and approved by the Institutional Ethics Committee for the Care and Use of Laboratory Animals at the Faculty of Medicine and Pharmacy of Fez, Sidi Mohamed Ben Abdellah University, under ethical approval number 05/2023/LERHS. All necessary measures were taken to minimize animal suffering and to ensure compliance with the principles of humane care and use of laboratory animals.

## Conflicts of Interest

The authors declare no conflicts of interest.

## Data Availability

The data that support the findings of this study are available from the corresponding author upon reasonable request.
